# An American Story of a Health System Dilemma: The Slave Health Deficit and Post-Traumatic Slavery Stress Disorder (PTSSD)

**DOI:** 10.1007/s40615-025-02570-y

**Published:** 2025-07-28

**Authors:** Rodney G. Hood

**Affiliations:** 1 W. Montague Cobb/NMA Health Institute, Washington, DC USA; 2 National Medical Association, Silver Spring, USA; 3 Multicultural Health Foundation, San Diego, CA United States

**Keywords:** Slave health deficit, Post-traumatic slavery stress disorder, Ptssd, Health disparities, Racism in medicine, Chronic toxic stress, White supremacy and healthcare, Black holocaust

## Introduction

The purpose of this discussion is to enhance understanding of the historical foundations of current racial and ethnic health disparities. A pivotal narrative within American history will be examined with a focus on centuries of chronic emotional and physical trauma experienced by individuals of Black ancestry. The descendants of enslaved Black individuals continue to experience the enduring ethno-historic trauma rooted in slavery, further exacerbated by the persistent legacy of systemic oppression and the sustained transmission of racialized ideologies of inferiority. These intersecting factors have resulted in profound and multifaceted social, physical, and psychological consequences [1]. This paper will also explore the intricate dynamics between the lasting effects of slavery and contemporary health inequities while introducing the concept of racialized social determinants of health (rSDOH) in the United States. Historically, targeted discriminatory policies created cycles of disadvantage, resulting in disproportionate concentrations of affected populations in contexts marked by poverty, inadequate education, and poor health outcomes. Current evidence indicates that Black Americans face some of the worst health outcomes among racial groups resulting in excess premature deaths, characterized by elevated rates of infant and maternal mortality, as well as reduced life expectancies. According to the Center for Disease Control (2023), the age-adjusted death rates reveal profound racial health disparities: overall rates are 750.5 deaths per 100,000 US population, with American Indian/Alaska Native populations experiencing the highest rates 1,277.7 for males; 920.3 for females. Black Americans follow closely with 1,151.6 for males; 753.6 for females, while in descending order, White, Hispanic, and Asian populations exhibit significantly lower rates [2]. This paper will explore some of the root causes for these health disparities within the American healthcare system.

Part I provides an overview of critical topics, including the origins of humanity, the employment of pseudoscientific theories that supported concepts of White supremacy and Black inferiority, as well as the weaponization of Christian doctrine that reinforced false scientific beliefs justifying the institution of slavery of indigenous populations. To comprehend the impact of ethno-historical trauma on contemporary health disparities, particularly among Black Americans, this section examines historical events and erroneous notions of human hierarchy. A key historic event discussed include the Transatlantic Slave Trade—often termed the Black Holocaust [3] which lasted several centuries. These factors have led to significant intergenerational trauma for African slaves and their descendants, manifesting as increased chronic diseases and premature deaths within today's Black population. Part II presents an analysis of the inequitable healthcare received by Black Americans during and after slavery, revealing contemporary health disparities characterized as the “slave health deficit” [4–9], a term that depicts the origin of current Black health disparities stemming from slavery. This discussion will acknowledge the role of Western physicians and scientists who propagated false racial concepts that supported Black inferiority and contributed to today’s slave health deficits. A clear definition of White supremacy will be proposed to understand how this ideology has perpetuated systemic racial and ethnic inequities in contemporary American society. Part III introduces the hypothesis of post-traumatic slavery stress disorder (PTSSD) [8, 10], which posits that intergenerational trauma from slavery results in persistent toxic stress, as measured by allostatic load, thereby contributing to adverse health outcomes in Black Americans. Current evidence will be presented to underscore colorism [11] as a discriminatory factor supporting the PTSSD hypothesis, particularly through an analysis of differential toxic stress across racial and ethnic groups via comparative mortality and cardiovascular risk assessments. This discussion will also highlight the significant roles of the National Medical Association (NMA), W. Montague Cobb Health Institute, and NAACP in addressing these health inequities. Moreover, recent data will illustrate the significant human toll of poor health outcomes and mortality, especially for Black Americans, alongside the substantial economic burden associated with ongoing health disparities in the United States.

## Part I

### Origins of Humanity

The genetic and paleontological scientific evidence for common origins of humanity is found on the African Continent. This was revealed with the successful unraveling of the human genome in 2001. Early Western scientific concepts of the origin of modern humans, or Homo sapiens, did not perceive all racial groups, other than Africans, being originated on the African Continent, but rather proposed a multi-regional theory, suggesting humans found on other Continents where more evolved, falsely concluding that only Africans evolved from apes. However, the palaeontologic and genetic evidence shows that approximately 270,000 years ago all modern humans began to evolve on the African Continent [12]. Approximately 70,000 years ago, groups of these early modern humans began migrating out of Africa, eventually populating every continent [12]. However, many early Western scientists refused to accept the evidence of the single region theory because it did not coincide with their false belief of a human hierarchy with the White race at the top. We now know modern humans are remarkably genetically similar—approximately 99.9% of our DNA is identical across all people with more genetic diversity within different racial groups than between racial groups with the greatest diversity found in the African populations. This means that the variation we observed in phenotypical traits such as skin color, hair type, and various physical features accounted for only 0.1% of our genetic makeup. Using a perspective that considers the origins of the human genome originating on the African Continent, it can be argued that the human genome is logically classified as the “African Human Genome.” Furthermore, this underscores an important point: that race, as we often discuss it today, is not a strict biological category. Instead, it is largely a social construct shaped by historical and cultural factors as well as indigenous societal biases. In essence, we can say that we are part of a single human race, with diverse ethnicities that have evolved from our shared ancestry in Africa, but false concepts of human hierarchy have created misconceptions and false beliefs of superior and inferior races amongst our human family. This discussion hopes to achieve a better understanding of our common origins and fosters a sense of unity among all people, despite the many differences that exist in appearances and cultures.

### Concepts of White Supremacy, Pseudoscience, Slavery, and Christianity

The racist concepts of White supremacy became rooted in American society as highlighted by the French philosopher Alexis de Tocqueville, with his personal observation of American society witnessed during his American journey from 1831–1832. Tocqueville’s Democracy in America journal on page 332 he writes [13], “Among these widely differing families of men, the first that attracts attention, the superior in intelligence, in power, and in enjoyment, is the white, or European, the MAN pre-eminently so called; below him appear the Negro and the Indian.………. we should almost say that the European is to the other races of mankind what man himself is to the lower animals: he makes them subservient to his use, and when he cannot subdue, he destroys them.”

The concept of White supremacy and pseudoscience has shaped our current understanding of humanity. If we take a step back in history, we find that many Western scientists, such as J.F. Blumenbach, played pivotal roles in promoting these flawed theories. In his 1781 work, On the Natural Variety of Mankind, Blumenbach suggested that the Caucasian skull represented the “most beautiful form” compared with the lesser extreme, the Ethiopians and Mongolians [5, 7, 14]. He even suggested that the white skin color must be the original skin color of humans. This idea not only positioned White individuals at the pinnacle of humanity but also set a dangerous narrative regarding people of color. There were individuals such as Carl von Linnaeus, considered Father of Biological Classification and Theophrastus Bombastus von Hohenheim—better known as Paracelsus—who contributed to the pseudoscientific belief that people of diverse races were somehow inferior to Caucasians. Linnaeus categorized humans into different “varieties,” each with its own set of traits that inherently framed non-White individuals as less civilized or intellectually capable. Paracelsus took that even further, proposing that Africans and other non-Christian groups descended from different, lesser ancestors, distancing them from the biblical Adam and Eve. Adding to this legacy of pseudoscience was Georges Cuvier (1769–1832), a prominent French naturalist and paleontologist, whose teachings influenced many American scholars [7, 14], such as Louis Agassiz, a professor of Medicine at Harvard University, and a key figure in American science, adopted similar views, promoting the idea of racial hierarchies based on perceived differences in intellect and moral character [14].

Another noted American physician who contributed to these false beliefs was Josiah Clark Nott, a graduate of the University of Pennsylvania School of Medicine, who founded the University of Alabama [5, 7, 14]. He not only practiced medicine but also owned slaves. He used his scientific standing to justify slavery itself, claiming “the negro achieves his greatest perfection, physical and moral, and greatest longevity, in a state of slavery.” Some scientists, such as Samuel George Morton (1799–1851) who taught at the University of Pennsylvania, promoted false disciplines of craniology and phrenology, which measured skull shapes and capacities, to argue larger skull capacities placed Whites at the top hierarchy and relegated Blacks to an inferior status—almost akin to apes on the evolutionary ladder [7, 14]. The belief in polygenesis or multi-regional theory of human origin—the idea that different races had separate geographical origins—was steadfastly adopted by most early Western scientists, although, this was directly contradicted by the scientifically supported view of monogenesis, which posits that all human beings share a common African ancestry. The early proponents of White supremacy refused to accept the evidence that points to Africa as the cradle of humanity from which all people evolved and those that did accept the single regional theory of human origin, continued to falsely postulate that human intellect and creativity only occurred after the populations migrated out of Africa.

The origins of White supremacy cannot be fully understood without considering the significant influence of Christianity. Numerous proponents of scientific racism have historically linked their unfounded claims of White superiority to various Christian beliefs and doctrines. For example, Johann Friedrich Blumenbach classified a skull discovered near the Caucasus Mountains in southeastern Europe as the epitome of beauty among human skulls, asserting its superiority over other racial forms, thus the origin of the name Caucasian to classify Whites [5, 7, 14]. After inspecting three mummies from ancient Egyptian catacombs, American scientist Samuel Morton concluded that Caucasians and Negroes were already distinct species three thousand years ago. Since the Bible indicated that Noah's Ark had washed up on Mount Ararat located in eastern Turkey, only a thousand years ago before this, Morton claimed that Noah’s sons could not possibly account for every race on earth. This association prompted some to assert that the biblical figures of Adam and Eve, as portrayed in Christian theology, were exclusively ancestors of Europeans, thereby excluding other racial groups from this narrative. Joshua Nott and other American scientists turned from their naturalists’ beliefs to their bible and deducted that Blacks were the children of Ham, son of Noah, who, along with his progeny, was “marked” and condemned to be servant to his brothers for having viewed his father’s nakedness [15].

This conflation of scientific inquiry with religious doctrine raises pertinent questions about its historical roots. A significant catalyst for this intertwining can be traced to the Christian Doctrine of Discovery, articulated in the mid-fifteenth century [16]. This doctrine, also referred to as the Doctrine of Domination, emerged during a period dominated by the exploration and colonization efforts of European powers, primarily Portugal and Spain, accompanied by a monolithic Christian Church prior to the Reformation. Pope Nicholas V issued the Doctrine of Discovery in 1452, which sanctioned the invasion and subjugation of non-Christian peoples and territories on the African Continent [16]. The decree explicitly stated that European Christians were permitted to “invade, search out, capture, vanquish, and subdue all Saracens (Muslims), and pagans whatsoever, and other enemies of Christ wheresoever placed.” Furthermore, it justified the appropriation of their possessions and the enslavement of individuals perpetually, all for the purpose of profit and the purported conversion to Christianity. This papal sanction suggested that the superior status of European Christians could be conferred upon the conquered populations through the introduction of Christianity, thus framing the appropriation of land and resources as a morally justified act. The Church subsequently issued various iterations of this Doctrine of Domination. In 1493, Pope Alexander VI issued the papal bull *interCaetera* that divided newly discovered lands between Spain and Portugal. This new doctrine provided moral and legal justification for Christopher Columbus’s return voyages to the New World in the Americas [16]. This effectively facilitated the establishment of Christian missions and contributed to the genocidal practices inflicted upon indigenous populations. White supremacy weaponized Christian doctrines to justify a racist social system constructed during the age of exploration, and played a crucial role in legitimizing acts of domination, exploitation, enslavement, and profoundly influenced societal attitudes toward race and superiority that persist in various forms today. This resulted in a social system with racist policies that guaranteed Blacks and Native Americans would be perpetual second-class citizens in America.

### The Black Holocaust and the Transatlantic Slave Trade

The topic of the Black Holocaust and the Transatlantic Slave Trade is a deeply painful and significant part of American history that continues to resonate today. It is crucial to acknowledge how false notions of White supremacy were used as a justification for the horrific treatment of both Native Americans and enslaved Africans over centuries. The Transatlantic Slave Trade with the enslavement of Africans in the Americas and Caribbean is referred to as the Black Holocaust. The Black Holocaust transpired over several centuries, and entailed a total death toll estimated between 6 to 150 million lives lost during and after the Transatlantic Slave Trade [3]. The United Nation estimates 17 million deaths while the World Future Fund estimates 60 million died during this span of time [3]. *The first period: —the Pre-transatlantic Slave Round-Up*, countless African men, women, and children were captured, forcibly taken to the western shores of Africa, and subjected to brutal conditions in dungeons. It has been estimated that between 6 to 10 million Black souls perished before they even set their feet on a slave ship. *The second period, the Transatlantic Voyage*, entailed the harrowing journey across the Atlantic, where enslaved individuals were crammed into ships under inhumane conditions. As many as 800 people were crammed together in the cargo holds of ships resulting in death rates reaching 20%. This means that 1.2 to 2.4 million souls lost their lives during the Transatlantic crossing because of the unsanitary and inhumane conditions they faced. Finally*, the third period, the Post-transatlantic Voyage Break-in Period*, in which the horrible living conditions and poor treatment did not end upon arrival. Within just 3 years, death rates for these new arrivals could soar to 30% or even 50%, as they faced the daunting challenge of acclimating to harsh new environments [5, 14]. It is notable that physicians were intimately involved in all three periods and served on slave ships to protect the cargo of slaves. Slave ship physicians’ salaries were reported to be second only to the captain of the ship, and a lucrative job for physicians was on plantations treating slaves.

In 2000 and 2002, authors W. Michael Byrd, MD, MPH and Linda Clayton, MD, MPH, in their extensively documented books, titled, An American Health Dilemma, Vol I and II [5, 14], revealed the significant impact of the pseudoscience of White supremacy on the poor health of American slave descendants. The authors documented statistics showing how the health of Blacks in America has been the worst compared to other racial groups, dating back to slavery, and highlighted the term, “slave health deficit,” a term coined by medical historians Dr. Todd L. Savitt and Dr. James O. Clayton, to describe the severe health disparities experienced by enslaved Africans in the United States [4–9]. Deficit refers to the negative health outcomes that result from unhealthy living and working conditions during and after slavery. Among the ten leading causes of death in the US, such as heart disease, cancer, stroke, diabetes, unintentional injuries, infant mortality, and maternal mortality, African slave descendants suffer the highest chronic disease and death rates. These racial health disparities can be less, depending on individual social and economic status, but race and ethnicity remain persistent independent risk factors for poor health outcomes. US health data clearly reveal Blacks have poor access to quality health care, and once they have accessed the health system, Blacks commonly are treated differently because of discriminatory institutional policies, as well as healthcare provider conscious or unconscious biases, which have been documented in many scientific publications on this subject [17–20].

How and why is this unique American experience by African slave descendants impacting us today? Information that will be presented later in this paper will highlight data that support the hypothesis of post- traumatic slavery stress disorder (PTSSD) and potential remedies for healing the affected populations.

## Part II

### Healthcare and Slavery in America

Slavery has been a part of human history for centuries, but the system established in the Americas and the Caribbean was particularly harsh and dehumanizing. Enslaved individuals were treated as valuable property, and as such, plantations hired physicians, often referred to as slave doctors, to meet their needs. Unfortunately, these medical practitioners adhered to the same biases and misguided beliefs as their European counterparts, viewing Black individuals through a lens of perceived inferiority. They often failed to connect the health issues faced by enslaved people to the brutal conditions and environments imposed upon them. The disadvantaged status of slaves created a large pool of individuals for medical experimentation, which allowed physicians to refine their treatments and techniques on enslaved bodies before applying them to their White patients. A notorious example is J. Marion Sims (1813–1883) who is often referred to as the “Father of Gynecology.” Sims conducted numerous surgical procedures on enslaved women without anesthesia, operating under the false belief that Black individuals possessed a greater tolerance for pain [5, 7, 15, 17]. This misconception has persisted through the years, with contemporary studies demonstrating that Black patients frequently receive inadequate pain relief compared with their White counterparts [17, 20]. Decades of unethical treatment and experimentation including the Tuskegee Syphilis study as documented by author, Harriett Washington in her book, Medical Apartheid [15], has created a significant distrust of the US health system for Blacks and other populations. This distrust further hinders the ability to effectively address the barriers of eliminating health disparities in these most affected populations.

Samuel A. Cartwright (1793–1863) was a graduate of the University of Pennsylvania School of Medicine who theorized that Blacks should medically be treated differently owing to their “nonhuman” biological peculiarities [6, 14, 21, 22]. Cartwright later became a professor of Medicine at the University of Louisiana. In 1851, he published a review article in a New Orleans medical journal called DeBow’s review titled “Diseases and Peculiarities of the Negro Race [19].” He coined the names of mental diseases that he attributed specifically to Blacks. He suggested that slaves who ran away from the brutality experienced on plantations suffered from a mental disease, *Drapetomania,* defined as runaway madness, and *Dysesthesia Aethiopica,* defined as uppity, rebelliousness or rascality. He prescribed treatment for drapetomania with condescension and brief periods of play time as though they were children. However, for those slaves that had dysesthesia aethiopica and continued to rebel, he prescribed whippings as punishment to “beat the devil out of them” and therefore prevent them from absconding.

### Post Slavery and Jim Crow

The post-slavery and Jim Crow eras in the United States are characterized by significant systemic inequities that have had lasting effects on African American communities, particularly in terms of access to healthcare, education, lack of social justice, and generational wealth. The 14th Amendment, ratified on June 13, 1866, was intended to secure citizenship for all individuals born or naturalized in the United States, including formerly enslaved individuals. Despite this, the implementation of Jim Crow laws entrenched a status of second-class citizenship, which persisted not only in Southern United States but also in various parts of the North. This institutionalized segregation, including redlining which limited where Blacks could live, fostered an environment where access to educational, healthcare, and financial resources for African Americans were vastly inferior.

During the early twentieth century, there were 7 medical schools designated for Black students; however, following the 1910 Flexner Report, a significant reduction occurred, leading to the closure of most of these institutions, leaving only Howard and Meharry Medical Schools operational [5, 23]. The ramifications of this action, combined with the limited financial resources available to the Black population and inadequate educational opportunities at historically Black colleges and universities (HBCUs), have contributed to a persistent shortage of Black physicians. According to recent data, while Black individuals make up approximately 13.7% of the overall US population, they account for only 5.7% of practicing physicians. Access to medical care for Black individuals was further hindered by the stark realities of segregated healthcare facilities. In many cases, hospitals that accepted Black patients maintained segregated wards, where they were typically treated by White physicians who often had exclusive admission privileges due to the organized medical community’s institutional racism, notably within the American Medical Association (AMA). The AMA's longstanding refusal to accept Black physicians severely restricted their ability to practice medicine and provide care within hospital settings. In 1893, John Hopkins University Hospital in Baltimore, Maryland opened with rigidly segregated classes, hospitals, and medical staff and remained segregated well into the 1960 s [5]. The desegregation of medical institutions, catalyzed by the Civil Rights Movement of the 1960 s, began to dismantle these barriers. Despite these advancements, the legacies of segregation and discrimination continue to resonate within healthcare systems today, leading to enduring health disparities.

The intellectual landscape of the nineteenth century further complicated the status of African Americans, as prominent academic figures propagated notions of racial inferiority. For example, Nathaniel Shaler at Harvard University espoused theories that positioned Black individuals as evolutionary inferiors, even justifying the violence of lynching as a misguided but noble attempt to protect societal values [15]. Similarly, Joseph LeConte’s writings at Harvard and later the University of California, reinforced the idea that slavery served a developmental purpose for the so-called “negro,” advocating for the denial of citizenship and voting rights in service of a paternalistic notion of protection and guidance. The post-slavery era and the Jim Crow period created a complex interplay of legal, educational, and healthcare inequities that profoundly affected African American communities, echoing through generations, and impacting contemporary structures. The historical context provided by White academic elites and the ingrained false assumptions that Blacks exhibited traits and tendencies due to their inferior status [24], only served to justify and sustain these inequities, contributing to the systemic challenges faced by Black Americans in the present day.

### Proposed Definition for White Supremacy in Healthcare

The late Walter Shervington, M.D., a notable psychiatrist, and the 99th President of the National Medical Association (NMA), posited that racism, particularly in its manifestation as White supremacy, ought to be regarded as a mental health disorder. He was unable to pursue his goal to advocate for this classification during his lifetime because of his untimely death. In 1969, a group of Black psychiatrists, many of whom belonged to the NMA, urged the American Psychiatric Association (APA) to acknowledge racism as a major mental health problem in this country and to recognized racism and bigotry as a mental illness in the Diagnostic and Statistical Manual of Mental Health (DSM), but this recommendation was rejected by the APA. However, I believe the idea has merit and warrants thoughtful exploration. Considering historical evidence and the behaviors exhibited by proponents of White supremacy, it is reasonable to propose a formal definition for this phenomenon. I suggest the term, Dysesthesia Caucasoid Myopia (DCM-Whitesupremacy), as a clinical identifier that deserves a DSM-5 definition and classification. The proposed mental disorder, DCM-White supremacy, can be characterized as a collective narcissistic behavior with a mentality steeped in misplaced arrogance, and grounded in discredited scientific notions that promote a hierarchical view of humanity. The underlying psychodynamics of DCM-White supremacy involve a subconscious inferiority complex that manifest as exaggerated self-opinion and myopic views of non-White ethnicities. This inflated sense of self-worth is fueled by an inappropriate sense of grandeur, which perpetuates false beliefs regarding individual and collective superiority. These distorted views not only harm those who are subjected to such ideologies but also lead to the systemic creation and enforcement of laws, policies, and social beliefs that prioritize the enrichment and advancement of White individuals at the expense of other racialized groups. Classifying White supremacy in this manner acknowledges the mental health implications of such beliefs and provides a framework for understanding their impact on society.

## Part III

### Post-Traumatic Slavery Stress Disorder (PTSSD)

Post-traumatic stress disorder (PTSD) is a psychiatric disorder that occurs in individuals who have experienced or witnessed a traumatic event, series of events or set of circumstances. Some researchers have linked the psychological residual effects of slavery with PTSD dysfunctional behaviors [25–29]. Clinical psychologist, Dr. Joy DeGruy introduced the concept of collective multigenerational trauma associated with centuries of pre and post slavery experiences in America and coined the term post-traumatic slavery syndrome (PTSS) [29]. This multigenerational trauma resulted in destructive behaviors that she described in 3 categories, vacant or low self-esteem, ever present anger, and racist socialization or internalized racism. Further research outlines how the impact of slavery and the oppression of African American people and their culture served as significant trauma to African Americans, which was carried forward through successive generations; providing an explanation of their current anxiety-related conditions, poor health, and maladaptive behaviors [26, 30]. These traumas resulted not only from the dehumanization experienced during slavery but post slavery Jim Crow Laws, terror of lynchings, and the intentional riots and massacres directed by Whites at economically successful Black communities, such as the New York City Draft Riots (1863), Colfax, Louisiana (1873), Wilmington, North Carolina (1898), Atlanta, Georgia (1906), Chicago, Illinois (1919), Elaine, Arkansas (1919), Tulsa, Oklahoma (1921), and Rosewood, Florida (1923), as well as and many other massacres [31, 32].

How is this past traumatic history related to the poor health outcomes for African Americans today? Recent compelling evidence suggests that these psychologically adaptive behaviors can lead to significant physical health consequences and chronic diseases later in life. I suggest we can expand this concept with the hypothesis of post-traumatic slavery stress disorder (PTSSD) [8, 10], which arises from the need to address the profound health effects of racism and the ethno-historical trauma experienced by descendants of American slaves. This hypothesis posits that “racism and the ethno-historical multigenerational trauma produced from the legacy of slavery in the United States, produces chronic physiological stress, measured as allostatic load, which significantly increases the risk for a variety of chronic diseases such as heart disease, hypertension, diabetes, infections, and cancer.” Collectively, these factors contribute to notable health disparities or slave health deficits, leading to early deaths within the US Black population. The trauma experienced over generations has had lasting effects—both psychologically and physiologically—on the descendants of enslaved Africans in the United States. This chronic form of stress manifests through complex mechanisms involving epigenetics, with research indicating that the traumatic experiences of past generations can influence the health outcomes of current generations [33, 34].

A critical component of this discussion is the concept of the allostasis which is defined as the physiological process by which the body achieves stability through change, adapting to various stressors encountered in the environment. In contrast, *allostatic load* refers to the cumulative physiological burden or *wear and tear* placed on the body because of repeated or prolonged activation of stress response systems, often termed chronic toxic stress. The allostatic load can be assessed through various biomarkers, including stress hormones and neurotransmitters. Daily exposure to racism contributes to this toxic stress, leading to an overproduction of biomarkers such as cortisol and epinephrine, and resulting in a decline in immune functions, for example, the suppression of immunoglobulin A (IgA). Furthermore, shortened telomere length associated with chronic stress has been linked to cell damage and decreased life expectancy [35]. Research supports the notion that Black individuals in the US have higher levels of stress biomarkers compared with their counterparts of other ethnicities, as well as a higher overall allostatic loads. A pivotal study published in the American Journal of Public Health in 2006 highlighted that Black individuals, particularly Black women, exhibit significantly elevated allostatic loads compared with White individuals, with even higher allostatic loads than their low-income White peers [36].

In addition, Adverse Childhood Experiences (ACEs)—stressful incidents encountered during childhood, such as parental incarceration or various forms of abuse—play a crucial role leading to diseases later in life. Higher ACE scores are consistently correlated with the emergence of chronic physical and mental illnesses later in life [37]. These experiences are believed to affect individuals through a combination of dysfunctional behaviors and altered stress-related biomarkers, impacting various bodily systems and the immune response. The prevalence of ACEs among Black children is striking. National statistics indicate that 61% of Black children faced at least one ACE, in contrast to 51% of Hispanic children, 40% of White Children, and 23% of Asian children [38]. The unique American cultural experience for Black individuals subjects them to various forms of racism—structural, personally mediated, and internalized [39–41]—leading to a heightened risk for PTSSD, which in turn contributes to chronic disease and premature mortality.

The concept of post-traumatic slavery stress disorder encapsulates the multifaceted impacts of historical trauma on the health of African American populations, emphasizing the need for a holistic understanding of the intersection between racism, psychological trauma, and physical health. Considerable information has been published in books and articles describing the relationship between stress and racism and its impact on health [21, 29, 40, 41]. It is imperative that we consider these factors when addressing the health disparities faced by Black individuals in the United States today.

### The Differing Effects of Chronic Toxic Stress Between Ethnic Populations in the United States

Chronic toxic stress as measured by the allostatic load is relevant across diverse human populations, though its impact and implications can vary widely among different racial demographic groups. The relationship between chronic toxic stress and health disparities is particularly evident in racial demographic statistics regarding beginning-of-life and end-of-life outcomes, as illustrated in Table 2. This data indicates African Americans experience the highest rates of infant and maternal mortality along with the shortest life expectancies. This is followed by American Indians/Alaska Natives (AI/AN), Whites, Hispanics, and Asians respectively. These findings suggest racial and ethnic groups suffer adverse health consequences that are contingent upon the duration and intensity of chronic stress because of their experiences with systemic oppression in the context of White supremacy.

A critical examination of historical factors reveals that the observed disparities may stem from a legacy of systemic dehumanization and cultural erasure that African Americans and Native Americans have endured over centuries. African Americans were subjected to the brutality of slavery, followed by the systemic oppression of Jim Crow laws and persistent institutional racism, despite the formal abolition of segregation. Similarly, Native Americans were forcibly removed from their ancestral lands, stripped of their cultural identities, and subjected to practices that led to significant population decline. Notably, Native American children were often placed in Eurocentric educational institutions which restricted the use of their native languages and the practice of their cultural customs.

During the period of slavery, African Americans were systematically denied literacy, depriving them of access to their historical narratives and severing ties to their cultural heritage. Post-emancipation, educational curricula predominantly focused on Eurocentric perspectives, further alienating African Americans from their identities. In contrast, while Hispanics and Asians have also encountered considerable oppression and discrimination, their experiences may lack the chronic depth and continuity of trauma faced by African Americans and Native Americans. These groups to varying degrees of emotional and physiological advantage, have managed to preserve elements of their cultural identities, such as language and customs. It is also essential to consider the chronic toxic stress experienced by the dominant White population, which may stem from an inherent fear of losing their societal status and privileges constructed upon the false and inhumane tenets of White supremacy. The ideology of racial superiority, historically perpetuated by the White elite, has facilitated the implementation of policies that harm marginalized populations. This fear-induced stress may contribute to less-than-optimal health outcomes for individuals within the oppressor class, particularly when compared to the health outcomes of Hispanic and Asian populations.

The socio-cultural framework of American society, underpinned by a doctrine of racial superiority, has created a toxic environment that adversely affects the health of both subordinate and dominant groups suggesting that current American culture can be bad for our health and wellbeing. This observation may partially explain the “immigrant health paradox” where immigrants are healthier compared to their native-born peers of similar demographics and socioeconomic profile. However, this paradox disappears as immigrants stay longer in the host country [42].

### Amount of Melanin in a Population and the Severity of Health Disparities

The relationship between melanin content in a population and the prevalence of health disparities is a multifaceted and underreported issue that merits thoughtful examination. Observations indicate that populations historically subjected to systemic discrimination and dehumanization, notably US Blacks and Native Americans, along with certain Hispanic sub-populations such as Puerto Ricans and Asians with South Asian ancestry, present higher rates of health disparities and cardiovascular disease risk than other populations with less African ancestry. White supremacy has led to a form of discrimination based upon one’s skin color referred to as *colorism* [11], which is prejudice or discrimination against individuals with dark skin tones. This observation makes it fair to say, that there is a direct correlation between the amount of melanin in a population and the severity of their health disparities.

This observation is confirmed when utilizing the Atherosclerotic Cardiovascular Disease (ASCVD) 10-year risk estimator (CV risk estimator) adopted by both the American Heart Association (AHA) and the American College of Cardiology (ACC) [43], which includes race and ethnicity as risk factors. When I introduced this health risk tool into my clinical practice, I encountered a 50-year-old African American male patient with the following cardiovascular (CV) risk profile: His blood pressure was recorded at 160/90, with a total cholesterol of 230, LDL cholesterol of 110, and HDL cholesterol of 45. Notably, he was a nonsmoker, had diabetes, and was currently on treatment for hypertension. His estimated 10-year CV risk was calculated at 25.1%, which was significantly above the optimal CV risk of 3.9%. In a striking comparison, when I then adjusted the ethnicity in his profile to identify him as a White male or categorized him as “Other Ethnicity,” with the same CV risk profile, the risk decreased to 15.1%. Moreover, this template revealed that a hypothetical 50-year-old Black female with the same risk profile, presented the highest cardiovascular risk of 27.2%, whereas a 50-year-old White female with a CV risk of 7.7% presented the lowest risk. Table 1 reveals for both the 40-year-old and 50-year-old, the Black female CV risk is greatest, followed by Black male > White male, and > White female. This increased CV risk for 40-year-old and 50-year-old Black female is supported by studies showing Black females have higher allostatic loads than Black males and as well as all other races and ethnicities [36].

**Table 1:** Compares ASCVD 10-year risk for hypothetical 40-year-old and 50-year-old Black and White male and female patients with same risk profile utilizing the ACC/AHA CV 10-year risk calculator

ACC/AHA 10-year ASCVD risk estimator (CV risk estimator) CV risk by race, ethnicity, and gender Table 1						
Diabetes Mellitus = yes Treatment HBP = yes Systolic BP = 160 Diastolic BP = 90 Total Cholesterol = 230 LDL Chol = 110 HDL Chol = 45 Cigarettes = no						
Racial Ethnic Subjects	Black American 10-year CV risk		White American 10-year CV risk		Disparity Ratio	
CV risk Disparities Ratio						
Age/Sex	Male	Female	Male	Female	Male	Female
40-yr-old	15.3%	20.3%	5.7%	4.6%	2.68	4.41
50-yr-old	25.1%	27.2%	15.0%	7.7%	1.66	3.53

Important to note, the ACC/AHA 10-year CV risk assessment tool may underestimate cardiovascular risk for certain “other” ethnicities, particularly American Indians, some Asian Americans of South Asian ancestry, and specific Hispanic groups, such as Puerto Ricans. Conversely, for individuals of East Asian ancestry and Mexican Americans—who generally have less African ancestry—risk assessments may be overestimated. These findings reinforce the literature indicating that Blacks, especially Black females, experience greater allostatic loads of chronic toxic stress, compared with all other races and ethnic groups [36]. These data suggest an urgent need to further investigate and address the root causes of these disparities. The interplay of race, ethnicity, and health should be critical areas for ongoing research and intervention within clinical practice.

When we look at cardiovascular (CV) risk, it is fascinating how disparities really pop out—especially for populations with significant African ancestry or those who have faced long-standing second-class status owing to systemic racism. These groups tend to have higher CV risks, sometimes just behind those of African Americans. This interesting observation seems to reveal a direct connection between melanin levels and colorism-based health disparities. This raises the following question: How much of this risk is tied to lived experiences, such as racism and discrimination? Now that we can measure levels of a person’s perceived racism, we have found that racism can make us sick [40, 41]. The ACC/AHA 10-year ASCVD risk estimator, which uses race and ethnicity as variables, may reflect the cardiovascular risk associated with heightened allostatic loads resulting from chronic stressors linked to discriminatory practices endemic in American society. While there have been advancements toward developing a CV risk calculator that does not incorporate race as a factor, but utilizes other social determinants, it is essential to note that the ACC/AHA algorithm has been validated as a reliable predictive tool across diverse populations, specifically for both Black and White cohorts.

The efficacy of the ACC/AHA 10-year CV risk tool has been corroborated through rigorous evaluation in several large Black cohorts, such as the Jackson Heart Study (JHS) and the Multiethnic Study of Atherosclerosis (MESA), demonstrating its predictive capabilities in these Black populations [44]. The observed disparities in cardiovascular (CV) risk highlight populations with significant African ancestry or those who have endured prolonged periods of racism and socioeconomic disenfranchisement exhibit increased CV risks, often second only to African Americans. I believe these observations shed light on how race and historical context can influence health outcomes and underscores the necessity of considering both biological and historical sociocultural factors in the assessment of cardiovascular risk. This aligns with research demonstrating a direct correlation between melanin concentration and the extent of health disparities within various populations with higher levels of African ancestry.

### Impact of the Slave Health Deficit on the Beginning-of-Life and End-of-Life

Throughout the twentieth century and into the twenty-first century, African Americans consistently faced higher mortality rates due to a significant number of leading causes of death. This persistent inequity extends beyond health and infiltrates economic, educational, and judicial systems, perpetuating cycles of disadvantage. However, the impact of these disparities is stark when we look at beginning-of-life and end-of-life data by examining key metrics such as infant mortality rates (IMRs), maternal mortality rates (MMRs), and overall life expectancy (LE). Data shows that in 1850, life expectancy was approximately 25.5 years for Whites and 21.4 years for Blacks. By 1900, Whites had an average life expectancy of 47 years, compared with 33 years for Blacks (Table 2). Fast forward to 2000, and while both groups saw improvements—White life expectancy reaching 77.6 years and Black life expectancy rising to 71.8 years—a gap of 5.8 years remains (Table 2). Additionally, maternal, and infant mortality rates consistently demonstrate that Black women and their infants experience the highest rates of morbidity and mortality compared with all other ethnic groups. A recent analysis published in June 2023 by the Journal of the American Medical Association (JAMA), highlighted that between 1999 and 2019, while overall maternal mortality rates saw some improvements, those for Black, American Indian, and Alaskan Native women significantly increased [45]. Although infant mortality rates improved during this period, they remained the highest for Black and American Indigenous infants (Table 2) [46, 47]. The ongoing health disparities faced by African Americans and other historically marginalized populations, highlight the entrenched nature of racialized inequities within the US healthcare system.

**Table 2:** Beginning-of-life and end-of-life mortalities and impact from COVID-19[45–49]

Centuries of Life Expectancy Disparities/End-of-Life Mortalities Table 2									
	Measure	Blacks	Dispar	Whites	Dispar	AI/AN	Hispanic	Asian	All
1850	Life Expectancy	21.4 yrs	−4.1	25.5 yrs	NA	NA	NA	NA	NA
1900	Life Expectancy	33 yrs	−14	47 yrs	NA	NA	NA	NA	NA
2000	Life Expectancy	71.8 yrs	−5.8	77.6 yrs	−4.5	73.1 yrs	78.8 yrs	85.7 yrs	76.9 yrs
Impact from the COVID-19 Pandemic									
2019	Life Expectancy	74.7 yrs	−4.1	78.8 yrs	−7.0	71.8 yrs	81.8 yrs	85.6 yrs	78.8 yrs
2020	Life Expectancy	71.7 yrs	−5.9	77.6 yrs	−10.5	67.1 yrs	78.8 yrs	83.5 yrs	77.3 yrs
Covid	*LE Difference	3.0 yrs	−1.8	1.2 yrs	−3.5	4.7 yrs	3.0 yrs	2.1 yrs	1.5 yrs
20 Years Persistent IMR Disparities and Worsening MMR Beginning-of-Life Mortalities									
1999	**MMR	31.4	−22.2	9.2	−9.8	19.0	9.6	9.6	12.7
	***IMR	14.0	−8.2	5.8	−3.5	9.3	5.7	4.8	7.00
2019	**MMR	67.6	−39.7	27.9	−41.4	69.3	20.8	20.8	32.2
	***IMR	10.62	−6.13	4.49	−3.38	7.87	5.03	3.38	5.58

*Life expectancy at birth differences 2019–2020 – (CDC – National Center for Health Statistics

** Maternal mortality rate (MMR) = deaths per 100,000 live births (JAMA, July 3, 2023)

*** Infant mortality rate (IMR) = deaths per 1000 live births

### Impact of the COVID-19 Pandemic

The COVID-19 pandemic has had profound and far-reaching impacts across the United States, with data from the Centers for Disease Control and Prevention (CDC) highlighting significant disparities among different racial and ethnic groups. The statistics reveal a concerning trend: Black, American Indian/Alaskan Native (AI/AN), and Hispanic populations faced disproportionately higher rates of COVID-19 infection and mortality compared with their White counterparts. In 2020, COVID-19 emerged as the third leading cause of death in the US, accounting for approximately 11% of all fatalities that year. According to CDC statistics reported by APM Research Lab [48], in December 2020, the cumulative COVID-19 mortality rates/100,000 by race and ethnicity, revealed Indigenous and Black individuals bore the brunt of this crisis, with death rates of 133 for Indigenous and 123.7 for Blacks. This was higher than death rates for Pacific Islanders (90.4), Hispanics (86.7), Whites (75.7), and Asians (51.6).

Furthermore, the pandemic contributed to a notable decline in life expectancy across the nation. The National Center for Health Statistics (NCHS) reported a decrease of 1.5 years in life expectancy for the total US population, dropping from 78.8 years in 2019 to 77.3 years in 2020 [49]. This decline is the most substantial 1-year reduction observed since World War II, bringing life expectancy to its lowest point since 2003. However, as depicted in Table 2, the impact was not uniformly distributed across racial and ethnic groups. Hispanics and non-Hispanic Blacks experienced a significant decline of 3.0 years in life expectancy in contrast to Whites with a decline in life expectancy of 1.2 years and later obtained data for AI/AN and Asians showed a decline in life expectancy of 4.7 years and 2.1 years respectfully as shown in Table 2. These disparities underscore the longstanding issues that historically marginalized populations face, particularly during crises. The often-used phrase, “When White folks catch a cold, Black folks catch pneumonia,” captures the essence of this reality. During catastrophic events such as pandemics, wars, or natural disasters such as seen with Hurricane Katrina, marginalized communities frequently find themselves at a disadvantage, facing significant healthcare disruptions, economic instability, structural racism, and increased social isolation.

### The Cost of US Health Inequities

A recent study sponsored by the National Institute of Health (NIH) and published in JAMA in 2023 highlighted the significant burden of health disparities affecting the Black population in the United States. Over a 22-year period, from 1999–2020, the data reveal that this group experienced more than 1.63 million excess deaths compared with their White counterparts, along with more than 80 million excess years of potential life lost [50]. In a related finding, another study published in May 2023 by JAMA, addressed the economic implications of these health disparities. It is estimated that in 2018 alone, the cost of racial and ethnic health disparities to the US economy was a staggering $451 billion—a substantial 41% increase from estimates provided in 2014. Notably, a significant portion of this economic burden fell predominantly on the Black/African American population, accounting for 69% of the costs attributed to premature mortality [51].

These findings underscore the urgent need to address the systemic issues contributing to health inequities. There is not only a moral imperative to address these excess death disparities, but a financial opportunity to capture significant cost savings for an already expensive US health system.

### Social Determinants of Health (SDOH) Barriers to Achieving Health Equity

The enduring pre and post slavery framework of racial hierarchy and the consequent discrimination against specific racial groups have led to the concept of racialized social determinants of health (rSDOH). Social determinants of health (SDOH) are non-medical factors that critically influence health outcomes, including the conditions of one’s birth, growth, work, and aging. In contrast, racialized social determinants of health (rSDOH) arise from systemic targeted racial discrimination through policies that disproportionately disadvantage the targeted racial groups. Historical practices such as Jim Crow laws, redlining, and segregated healthcare facilities have perpetuated these inequities, creating cycles of economic disadvantage leading to greater levels of poverty, particularly for Black and Native American communities. Recent data from the Kaiser Family Foundation (KFF-2023) reveal significant disparities in poverty rates among different racial groups in the United States, with American Indian/Alaskan Native individuals at 24.2%, Black individuals at 20.6%, Hispanic individuals at 16.6%, Asian/Pacific Islander individuals at 10.0%, and White individuals at 9.5% [52]. These statistics highlight the disproportionate levels of poverty with resultant barriers, faced by Black and Native American populations, to accessing educational opportunities, accumulating generational wealth [53], receiving quality healthcare with poor health outcomes [17, 54, 55], and avoiding premature mortality [50]. Effective policy formulation must not only acknowledge the impact of rSDOH but also work to dismantle barriers hindering equitable health outcomes for these populations.

### The Role of the National Medical Association (NMA) and Health Equity

The National Medical Association (NMA) has played a pivotal role in addressing health equity, particularly for African American physicians and their patients. Founded in 1895 in response to systemic exclusion of Black physicians from the American Medical Association (AMA) and other medical groups, the NMA aimed to foster collaboration and support among Black medical professionals. According to the founding members, the original purpose remains relevant today, “Conceived in no spirit of racial exclusiveness, fostering no ethnic antagonisms, but born of the exigencies of the American environment, the National Medical Association has for its object the banding together for mutual cooperation and helpfulness, the men and women of African descent who are legally and honorably engaged in the practice of the cognate professions of medicine, surgery, pharmacy and dentistry.”

Leadership within the NMA, particularly figures such as past presidents W. Montague Cobb (1964) and John L.S. Holloman (1966), strongly advocated for health and social equity. These leaders recognized that the broader social environment had serious health implications, especially considering the frequent lynchings. During the late nineteenth and early twentieth centuries, over 4,700 lynchings of mostly Blacks by Whites were documented, causing physical and emotional terror within the Black communities, and was identified as glaring life ending health disparities [56, 57]. In the early years of its founding, the NMA collaborated with other Black organizations such as the National Association for the Advancement of Colored People (NAACP), to end lynchings against Blacks throughout America. In fact, the NMA changed its planned national meeting from Memphis, Tennessee to Philadelphia to protest the lynching of Ell Persons on May 22, 1917 [56, 57]. These groups advocated enacting anti-lynching laws but were always blocked by racist members of the US Congress.

Dr. Cobb also served as the NAACP president from 1976–1982 and encouraged Black physicians to financially support this organization, and with Dr. Cobb’s leadership, the NAACP served as an action arm of the NMA. In the 1960 s, the NMA was the only medical organization in support of Civil Rights legislation, leading to the desegregation of hospitals and White medical organizations as well as the implementation of Medicare and Medicaid. The AMA opposed this legislation characterizing it as socialized medicine. However, its passage resulted in drastic improvements in access for underserved populations, especially for African American patients, who receive better quality health care with improved health outcomes.

Although health disparities are believed to be caused primarily by poor social and economic conditions, referred to as social determinants of health (SDOH), the role of racism in medicine was rarely mentioned or minimized as a cause of these disparities. However, many members of the NMA believed that racism played a significant role in contributing to persistent health inequities, leading to health disparities. In the late 1990 s, because of NMA health policy advocacy, working with the Congressional Black Caucus (CBC), the United States Congress funded the Institute of Medicine (IOM) and National Academy of Science report published in 2002, *Unequal Treatment: Confronting Racial and Ethnic Disparities in Healthcare* [17]*.* This landmark report documented structural racism and healthcare provider biases that contributed to health disparities, especially in Black populations. Since then, hundreds of scientific publications have independently confirmed that racism, race, and ethnicity are independent variables and significant contributors to racial health disparities, specifically, the slave health deficit.

The NMA, as well as its affiliate, the W. Montague Cobb/NMA Health Institute, have been instrumental in both recognizing and addressing the complex interplay between race, health, and social determinants. For over 20 years, the Cobb/NMA Health Institute has focused research on racial and ethnic health disparities, developing clinical-community linkages to reduce health disparities. Two scientific journals are published to support this mission, including the Journal of the National Medical Association (JNMA), published by the NMA, and the Journal of Racial and Ethnic Health Disparities (JREHD) published by the Cobb/NMA Health Institute. As research continues to affirm the impact of racism as an independent variable in health disparities, the ongoing work of the NMA and the Cobb Health Institute remains crucial in the pursuit of health equity for all.

### Remedies—Remove the Mask

W.E.B. Du Bois, in his seminal work “The Souls of Black Folk,” articulates a profound concept known as “double consciousness” [58]. This phenomenon describes the dual awareness that African Americans possess: they see themselves through the lens of a prejudiced White society while simultaneously striving to maintain their own unique Black identity. This internal conflict is deeply rooted in the legacy of slavery, where survival often necessitated the internalization of damaging beliefs perpetuated by a system of White supremacy, leading to entrenched forms of internalized racism [39, 55]. African slave descendants understood that revealing their true selves could result in severe repercussions, including, loss of income and acceptance. During slavery and Jim Crow, repercussions were more severe, such as whippings or even death, in the form of lynching.

In 1895, the African American poet, Paul Laurence Dunbar powerfully captured the pain of this conflict which was illuminated in his poem, “We Wear the Mask.”

We wear the mask that grins and lies, It hides our cheeks and shades our eyes, This debt we pay to human guile, With torn and bleeding hearts we smile. And mouth with myriad subtleties. Why should the world be over wise, In counting all our tears and sighs? Nay, let them only see us while we wear the mask. We smile, but O great Christ, our cries To thee from tortured souls arise. We sing, but oh the clay is vile beneath our feet, and long the mile, But let the world dream otherwise. We wear the mask.

The metaphor of the mask serves as a critical lens through which one can examine the intricate interplay between emotional expression and societal expectations. In the lines “We wear the mask that grins and lies,” Dunbar articulates the psychological burden associated with emotional concealment, particularly in the context of pain, masked by outward appearances, as evidenced by the phrase “With torn and bleeding hearts we smile.” This encapsulates a profound tension between the yearning to express one's authentic feelings and the pressure to conform to societal norms that often demand stoicism.

To facilitate removing the metaphorical mask with healing and reconciliation within society, it is essential to engage and acknowledge the historical truths that underpin these emotional experiences. Specifically, there is a pressing need to correct the historical inaccuracies that have been perpetuated within dominant narratives.

### Policy and Clinical Remedies

- *Medical Health Reparations* [6, 59, 60]—to repair and restore quality health status for the US Black populations we need adequate funding of Black health institutions such as HBCUs, mental health centers, and research focused on the epigenetic and intergenerational traumas of communities with inherited deficits.
- *Trauma-informed Care*—to address the psychological trauma associated with the dual consciousness present in the Black population along with the denial and apathy in the majority population, it is necessary to raise awareness and implement academic and community-based educational programs that incorporate healing principles, such as:
- *Critical consciousness* [61, 62]—to recognize how systems of privilege and oppression contribute to trauma disproportionately affecting marginalized communities.
- *Cultural humility* [63, 64]—learning to acknowledge the significance of cultural differences of others and engage in self-reflection to address one’s own biases.
- *Radical healing* [65]—to learn and address the root causes of trauma, particularly identity-based wounds stemming from racism and other forms of discrimination.
- *Restorative justice* [66]- to repair harm, restore relationships, and rebuild communities affected by trauma and conflict, empowering individuals, and promoting healing.
- Community Resilience Programs [67]—to collectively support culturally anchored stress-reduction initiatives such as faith-based wellness and historical healing circles.

Such programs must aim to broaden perspectives and promote transformative thinking. The pursuit of equity and justice within healthcare and broader societal domains is a collective responsibility that warrants concerted efforts and unwavering commitment from all individuals.

## Conclusion

This paper explores the historical and contemporary impacts of systemic discrimination and racism on African American descendants of enslaved individuals and Native American communities in the United States. It highlights intergenerational trauma and underscores the significance of recognizing cultural differences to achieve equitable health outcomes and enhance societal well-being. The health disparities observed in marginalized populations are rooted not merely in individual choices or socioeconomic conditions, but in centuries of institutional discrimination, systemic racism, and systematic inhumanity directed towards these groups. Such historical injustices have fostered significant ethno-historical trauma that manifests in toxic stress responses, adversely affecting health outcomes, contributing to chronic diseases, and increasing premature mortality.

Since the inception of the United States, a political and social hierarchy has favored White individuals, particularly affluent White males. The US Constitution historically conferred voting rights and governmental privileges exclusively on White men, systematically disenfranchising African Americans, Native Americans, women, and other marginalized groups. This exclusionary framework has been underpinned by a belief in White superiority, as critiqued through the concept of Dysesthesia Caucasoid Myopia, which reflects a narrow and myopic understanding adopted by those in privileged positions regarding the experiences of marginalized communities. A critical reevaluation of historical contexts is essential for fostering a more inclusive understanding of our collective past. The prevalent notion of a “color-blind” society obscures the persistent racial disparities and overlooks the adverse health outcomes stemming from the intersections of race, identity politics, and systemic oppression.

Further investigation into epigenetics and racial disparities is necessary to clarify how trauma and stressors perpetuate chronic conditions across generations. Data showing the children of chronically marginalized communities have higher ACE scores, especially Black children, suggest research is warranted into early intervention strategies to avoid later in life consequences. Additionally, there is a need for more research focusing on intergenerational chronic toxic stress and their connections to racial and social determinants of health (rSDOH). While African Americans and Native Americans disproportionately experience health inequities, it is also important to recognize that segments of the White population can suffer adverse health outcomes when societal complexities and cultural identities are homogenized. Data suggest that cohesive cultural identities, such as those found in Hispanic and Asian communities, often correlate with improved health metrics, underscoring the importance of cultural preservation and acknowledgment.

The loss of 1.63 million excess deaths among Black Americans, which equates to over 80 million years of excess life lost over two decades, can be likened to modern-day lynchings, fueled by a collective apathy toward racial health disparities. This critical health crisis should serve as a moral imperative for America to act. Economically, these health disparities represent an estimated annual excess cost of $451 billion, providing a compelling rationale for prioritizing solutions aimed at reducing health inequities. Addressing these disparities could alleviate a portion of the staggering $4.5 trillion annual healthcare expenditure in the US. The costs associated with racial and ethnic health disparities necessitate the development of transformative health equity initiatives, including medical reparations and trauma-informed care principles, which could be funded through projected savings from reduced disparities.

In summary, the systemic challenges at the intersection of race, identity, and health require a comprehensive reassessment of societal values and structures. The interplay of historical trauma, toxic stress, and health disparities necessitates urgent action to dismantle inequitable systems. By cultivating an environment that honors diversity, advocates for social justice, and engages in honest discourse about our collective history, we can pave the way for a more equitable and healthier society for all. This approach serves not only to address the critical health issues facing the American health system but also to rectify broader societal dysfunctions, emphasizing the crucial need for cultural affirmation.

## Data Availability

Not applicable.

## Data Availability

Meets criteria.

## References

[1] Akbar, N. (1996). Breaking the chains of psychological slavery. Tallahassee, FL: Mind Productions & Associates.

[2] Center for Disease Control and Prevention (CDC) (December 2024). National Center for Health Statistics (NCHS), National Vital Statistics System, Mortality Data File No. 521.

[3] World Future Fund. (2005). Death toll from the slave trade: The African Holocaust. Retrieved from https://worldfuturefund.org/Reports/Slavedeathtoll/slaverydeathtoll.html

[4] Byrd WM, Clayton LA. The “slave health deficit”: Racism and health outcomes. Health/PAC Bulletin. 1991;21:25–8. 10115982

[5] Byrd, W. M., & Clayton, L. A. (2002). An American health dilemma. Volume II: Race, medicine, and health care in the United States, 1900–2000. New York: Routledge; Blumenbach, pages 12–13, slave health deficit, pages 194–197, Flexner Report, pages 93–101, John Hopkins Hospital, page 15.

[6] Hood, R. G. (2001). The slave health deficit: The case for reparations to bring health parity to African Americans. Journal of the National Medical Association, 93(1). PMC2640620 12653374

[7] Hood, R. G., et al. (2001). Racism in medicine and health parity for African Americans – The slave health deficit. Presented at the Second Annual National Medical Association Colloquium on African American Health, Washington, DC. Reprint upon request.

[8] Hood, R. G. (2013-present). The ethno-historical legacy of slavery in America as a root cause for health inequities for African Americans, and post-traumatic slavery stress disorder (PTSSD). Unpublished multimedia presentation. Presentation copy upon request.

[9] Savitt, T. L. (1978; reprint 2002). Medicine and slavery: The diseases and health care of Blacks in antebellum Virginia. Urbana: University of Illinois Press.

[10] Hood, R. G. (2023). Post-traumatic slavery stress disorder (PTSSD). MD Newsline. Retrieved from https://youtu.be/nRyIY_UEsnc?si=C9xDStZIxAGQEVEu 10.1007/s40615-025-02570-y PMC12446405 40721709

[11] Bell, T. N. (2023). The Connection Between Colorism and White Supremacy: Colorism as a Tool of White Supremacy in the Contemporary Age in the United States and Puerto Rico. Senior Theses. Retrieved from https://research.library.fordham.edu/international_senior/115

[12] Map of human migration out of Africa. (2023). Retrieved from https://youtu.be/IcdRkeTb6gM.

[13] Tocqueville A. Democracy in America. Observation of American society. 1994;1831–1832:332.

[14] Byrd, W. M., & Clayton, L. A. (2000). An American health dilemma. Volume I: A medical history of African Americans and the problem of race: Beginning to 1900. New York: Routledge; J. Blumenbach, Natural Variety of Mankind, pages 215–216, Georges Cuvier, pages 98, 217–220, Louis Agassiz, pages 296–298, 301–302, J. Nott, page 299, George Morton, page 103–105, S. Cartwright, pages 299–300, break-in period, page 196, J.M. Sims, pages 271–276.

[15] Washington, H. A. (2008). Medical apartheid: The dark history of medical experimentation on Black Americans from colonial times to the present; J.M. Sims, pages 61–70, Nathan Shaler, page 39, J. Nott and Christianity, pg. 39.

[16] Charles, M., & Rah, S. C. (2019). Unsettling truths: The ongoing dehumanizing legacy of the Doctrine of Discovery.

[17] Smedley, B., & Stith, A. (2002). Unequal treatment: Confronting racial and ethnic disparities in health care. Institute of Medicine; Board on Health Sciences Policy. 25032386

[18] Green AR, et al. Implicit bias among physicians and its prediction of thrombolysis decisions for Black and White patients. JGIM. 2007;22(9):1231–8. 17594129 10.1007/s11606-007-0258-5 PMC2219763

[19] Sabin JA, et al. Physicians’ Implicit and Explicit Attitudes About Race by MD Race. Ethnicity, and Gender, J Health Care Poor Underserved. 2009;20(3):896–913. 19648715 10.1353/hpu.0.0185 PMC3320738

[20] Meghani SH, Byun E, Gallagher RM. Time to take stock: a meta-analysis and systematic review of analgesic treatment disparities for pain in the United States. Pain Med. 2012;13(2):150–74. 22239747 10.1111/j.1526-4637.2011.01310.x

[21] Williams, R.A. (2001). Humane medicine: A new paradigm in medical education and health care delivery, Vol II; Stress and Health, pages, 49–55, J. Cartwright, pages 36–37.

[22] Cartwright, S. (1851). Diseases and peculiarities of the Negro race. DeBow’s Review, Southern and Western States, 11. New Orleans: AMS Press, Inc. New York, 1967.

[23] Flexner, A. (1910). Medical education in the United States and Canada: A report to the Carnegie Foundation for the Advancement of Teaching. Reprinted 1972. PMC2567554 12163926

[24] Hoffman, F. L. (1896). Race, traits, and tendencies of the American Negro. The Lawbook Exchange.

[25] Hankerson SH, Moise N, et al. The intergenerational impact of structural racism and cumulative trauma on depression. Am J Psychiatry. 2022;179(6):434–40. 35599541 10.1176/appi.ajp.21101000 PMC9373857

[26] Halloran MJ. African American health and post-traumatic slave syndrome: A terror management theory account. J Black Psychol. 2019;50(1):45–65.

[27] Wilkins EJ, Whiting JB, Watson MF, et al. Residual effects of slavery: What clinicians need to know. Contemp Fam Ther. 2013;35:14–28.

[28] Wilkins EJ, Whiting JB, Watson MF, Russon J, Moncrief A. Residual effects of slavery: A Delphi study. Contemporary Family Therapy: An International Journal. 2022;44(3):234–43.

[29] DeGruy, J. (2005). Post Traumatic Slave Syndrome. Uptone publisher.

[30] Nobles WW. Natural/man-made disaster and the derailment of the African worldview. J Black Psychol. 2013;39:252–6.

[31] BET News: (2021). Not just Tulsa: Race massacres that devastated Black communities in Rosewood, Atlanta, and other American cities: https://www.bet.com/article/fqn50c/five-other-race-massacres-that-devastated-black-america

[32] Atlanta Black Star (Dec. 2013, updated 2019): 8 Successful and Aspiring Black Communities Destroyed by White Neighbors: https://atlantablackstar.com/2013/12/04/8-successful-aspiring-black-communities-destroyed-white-neighbors/

[33] Lehrner A, Yehuda R. Cultural trauma and epigenetic inheritance. Dev Psychopathol. 2018;30(5):1763–77. 30261943 10.1017/S0954579418001153

[34] Yehuda R, Lehrner A. Intergenerational transmission of trauma effects: Putative role of epigenetic mechanisms. World Psychiatry. 2018;17(3):243–57. 30192087 10.1002/wps.20568 PMC6127768

[35] Epel ES, Prather AA. Stress, telomeres, and psychopathology: Towards a deeper understanding of a triad of early aging. Annu Rev Clin Psychol. 2018;14:371–97. 29494257 10.1146/annurev-clinpsy-032816-045054 PMC7039047

[36] Geronimus A, Hicken M, et al. “Weathering” and age patterns of allostatic load scores among Blacks and Whites in the United States. Am J Public Health. 2006;96:826–33. 16380565 10.2105/AJPH.2004.060749 PMC1470581

[37] Felitti VJ, et al. Relationship of childhood abuse and household dysfunction to many of the leading causes of death in adults: The adverse childhood experiences (ACE) study. Am J Prev Med. 1998;14(4):245–58. 9635069 10.1016/s0749-3797(98)00017-8

[38] Sacks, V., & Murphey, D. (2018). Prevalence of adverse childhood experiences, nationally, by state, and by race or ethnicity. Child Trends, Report Child Welfare, February 12.

[39] Jones CP. Levels of racism: A theoretic framework and a gardener’s tale. Am J Public Health. 2000;90(8):1212–5. 10936998 10.2105/ajph.90.8.1212 PMC1446334

[40] Williams, D., & Mohammad, S. (2009). Understanding how discrimination might affect health. Journal of Behavioral Medicine.

[41] Williams, D. R. (2016). How racism makes us sick. TED Talk. Retrieved from https://youtu.be/VzyjDR_AWzE

[42] Bacong AM, Menjivar C. Recasting the immigrant health paradox through intersections of legal status and race. J Immigr Minor Health. 2021;23(5):1092–104. 33656653 10.1007/s10903-021-01162-2 PMC10022586

[43] American College of Cardiology. ASCVD 10-year Risk Estimator Plus, ASCVD Risk Estimator. https://tools.acc.org/ascvd-risk-estimator-plus/#!/calculate/estimate/

[44] Fox ER, et al. Development and validation of risk prediction models for cardiovascular events in Black adults: The Jackson Heart Study cohort. AMA Cardiology. 2016;1(1):15–25. 27437649 10.1001/jamacardio.2015.0300 PMC5115626

[45] Fleszar, L. G. (2023). Trends in state-level maternal mortality by racial and ethnic groups in the United States. JAMA. 10.1001/jama.2023.9043 PMC10318476 37395772

[46] Mathews, T. J. (2002). Infant mortality statistics from 1999 period. CDC National Vital Statistics Report, 5(4). 11837053

[47] Ely, D. M. (2021). Infant mortality in the United States, 2019: Data from period linked birth/infant death file. CDC National Vital Statistics Report, 70(14). 34878382

[48] APM Research Lab. (2020). The color of coronavirus: COVID-19 deaths by race and ethnicity in the U.S. Color of Coronavirus, 2020 in review.

[49] Arias, E., et al. (July 2021). Life expectancy declines between 2019–2020: Provisional life expectancy estimates for 2020. CDC National Center for Health Statistics, Report No. 015.

[50] Caraballo, C., et al. (2023). Excess mortality and years of potential life lost among the Black population in the U.S., 1999–2020. JAMA, 329. 10.1001/jama.2023.7022 PMC10189563 37191702

[51] LaVeist, T. A., Pérez-Stable, E. J., Richard, P., et al. (2023). The economic burden of racial, ethnic, and educational health inequities in the U.S. JAMA, 329(19), 1682–1692. 10.1001/jama.2023.5965 37191700

[52] Kaiser Family Foundation (KFF)-State Health Facts: The independent source for health policy research, polling, and news: Poverty Rate by Race/Ethnicity | KFF

[53] Plumer, B. (2013). These ten charts show the Black-White economic gap has not budged in 50 years. Retrieved from Washington Post: https://www.washingtonpost.com/news/wonk/wp/2013/08/28/these-seven-charts-show-the-black-white-economic-gap-hasnt-budged-in-50-years/?noredirect=on&utm_term=.0ec9624ef030

[54] Ehlers CL, Gizer IR, Gilder DA, Ellingson JM, Yehuda R. Measuring historical trauma in an American Indian community sample: contributions of substance dependence, affective disorder, conduct disorder, and PTSD. Drug Alcohol Depend. 2013;133(1):180–7. 23791028 10.1016/j.drugalcdep.2013.05.011 PMC3810370

[55] Mouzon DM, McLean JS. Internalized racism and mental health among African Americans, US-born Caribbean Blacks, and foreign-born Caribbean Blacks. Ethn Health. 2016;22:36–48. 27354264 10.1080/13557858.2016.1196652

[56] Equal Justice Initiative (2017). Lynchings in America: Confronting the legacy of racial terror. Montgomery, AL: Third edition.

[57] Allen, J. (2000). Without sanctuary: Lynching photography in America. Twin Palms.

[58] Du Bois, W. E. B. (1903). The Souls of Black folks.

[59] Robinson R. The Debt: (2000). What America Owes to Blacks. New York: Dutton Penguin Group.

[60] Williams DR, Collins C. Reparations: A viable strategy to address the enigma of African American health. America Behavioral Scientist. 2004;47:977–1000.

[61] Diemer MA, Rapa LJ, et al. Critical consciousness: A developmental approach to addressing marginalization and oppression. Child Development Perspectives. 2016;10(4):216–21.

[62] Mejia Y, Christophe NK, et al. Critical consciousness and anti-racist action as rooted in family processes. Fam Process. 2024;63(2):630–47. 38881163 10.1111/famp.13025

[63] Robinson, D., et al. (2021). The 5 Rs of Cultural Humility: A Conceptual Model for Health Care Leaders, The American Journal of Medicine, Volume 134, 161–163. 10.1016/j.amjmed.2020.09.029 33198950

[64] Foronda C, et al. Cultural Humility: A concept analysis. J of Transcultural Nursing. 2016;27(3):210–7. 10.1177/1043659615592677 26122618

[65] Neville, H.A., et al. The Psychology of Radical Healing Collective, The Psychology of Radical Healing: What can psychology tell us about healing from racial and ethnic trauma? Psychology Today, online. https://www.psychologytoday.com/us/blog/healing-through-social-justice/201903/the-psychology-radical-healing?msockid=1daf1c16618a63b53df50fd360186252

[66] Fulham L, et al. The effectiveness of restorative justice programs: A meta-analysis of recidivism and other relevant outcomes. Criminol Crim Just. 2023. 10.1177/17488958231215228.

[67] Kirby, N. (2025). Strengthening community resilience through participation – a conceptual exploration. Environmental Sociology, 1–16. 10.1080/23251042.2025.2479666

